# Disruption of the microbiota affects physiological and evolutionary aspects of insecticide resistance in the German cockroach, an important urban pest

**DOI:** 10.1371/journal.pone.0207985

**Published:** 2018-12-12

**Authors:** Jose E. Pietri, Connor Tiffany, Dangsheng Liang

**Affiliations:** 1 Apex Bait Technologies, Inc., Santa Clara, California, United States of America; 2 University of California, Davis School of Medicine, Department of Medical Microbiology & Immunology, Davis, California, United States of America; Institute of Plant Physiology and Ecology Shanghai Institutes for Biological Sciences, CHINA

## Abstract

The German cockroach, *Blatella germanica*, is a common pest in urban environments and is among the most resilient insects in the world. The remarkable ability of the German cockroach to develop resistance when exposed to toxic insecticides is a prime example of adaptive evolution and makes control of this insect an ongoing struggle. Like many other organisms, the German cockroach is host to a diverse community of symbiotic microbes that play important roles in its physiology. In some insect species, there is a strong correlation between the commensal microbial community and insecticide resistance. In particular, several bacteria have been implicated in the detoxification of xenobiotics, including synthetic insecticides. While multiple mechanisms that mediate insecticide resistance in cockroaches have been discovered, significant knowledge gaps still exist in this area of research. Here, we examine the effects of altering the microbiota on resistance to a common insecticide using antibiotic treatments. We describe an indoxacarb-resistant laboratory strain in which treatment with antibiotic increases susceptibility to orally administered insecticide. We further reveal that this strains harbors a gut microbial community that differs significantly from that of susceptible cockroaches in which insecticide resistance is unaffected by antibiotic. More importantly, we demonstrate that transfer of gut microbes from the resistant to the susceptible strain via fecal transplant increases its resistance. Lastly, our data show that antibiotic treatment adversely affects several reproductive life-history traits that may contribute to the dynamics of resistance at the population level. Together these results suggest that the microbiota contributes to both physiological and evolutionary aspects of insecticide resistance and that targeting this community may be an effective strategy to control the German cockroach.

## Introduction

The German cockroach, *Blatella germanica*, is a widespread urban pest of medical and economic importance. *B*. *germanica* is a known or suspected mechanical vector of numerous enteric bacterial pathogens [1–5] and is also a significant contributor to allergic asthma disease [6–7]. The saliva, feces and shed cuticle of the German cockroach contain several potent allergens that have been detected at concentrations associated with allergic sensitization and asthma morbidity in 10–13% of U.S. homes [8]. Due to growing concerns regarding the development of resistance to commonly used insecticides in field populations [9], the improvement of current tools for the management of cockroach infestations is an ongoing research priority.

The use of antibacterial treatments has the potential to facilitate control of a range of insects. Most arthropod species have evolved intricate relationships with symbiotic bacteria and depend on microbes for reproduction, development, metabolism, and immunity, to some extent [10]. Disrupting these commensal bacteria can have adverse effects on insect physiology resulting in death or reduced fitness. For example, direct mortality was observed in adult tsetse flies fed a blood-meal containing the antibiotic tetracycline as well as in lice fed four different antibiotics [11–12]. On the other hand, elimination of *Wigglesworthia glossinidia* from tsetse flies by oral provisioning of tetracycline reduced fecundity and pupal emergence of F1 offspring, while elimination of *Sodalis glossinidius* with streptozotocin reduced the longevity of the offspring [13]. Similarly, pea aphids fed rifampicin had shorter adult lifespans and reduced production of F1 offspring relative to controls [14]. In the omnivorous American cockroach, removal of symbiotic bacteria from the gut with metronidazole reduced weight gain in developing nymphs [15]. The removal of bacterial symbionts can not only affect insect lifespan and fecundity, but may also impact insecticide resistance [16]. Specifically, in both the bean bug, *Riptortus pedestris*, and oriental fruit fly, *Bactrocera dorsalis*, insecticide resistance has been attributed to the production of detoxification enzymes by Proteobacteria located in the midgut and antibiotic treatments can restore susceptibility to resistant individuals [17–18].

The German cockroach harbors a diverse microbiota. The primary cockroach symbiont, *Blattabacterium cuenoti*, is a vertically transmitted intracellular bacterium found at high titers in specialized cells of the fat bodies of all German cockroaches. During the nymph stage, the endosymbiont migrates to the ovaries and eventually becomes incorporated into developing oocytes, resulting in transmission from mother to offspring [19]. In addition, the gut microbiota of the German cockroach was recently characterized and found to be comprised primarily of Bacteroidetes, Firmicutes, Fusobacteria, and Proteobacteria [20]. This dynamic microbial community, which changes throughout development from nymph to adult, is thought to be acquired horizontally from the environment and diet, as well as vertically through the consumption of feces (coprophagy) from other members of a colony [20–23]. In both *B*. *germanica* and the related Panchlora cockroach, the microbial community also differs dramatically across sections of the gut [24–25].

Prior studies of multiple cockroach species have investigated the effects of the microbiota on host physiology, implicating commensal microbes in nutrient provisioning and metabolism, development, immunity, and aggregation behavior [15, 25–29]. However, the phenomenon of symbiont-mediated resistance has not been explored in *B*. *germanica*. Here, we sought to determine how the microbiota is involved in the development of insecticide resistance and the propagation of these traits through German cockroach populations using antibiotic treatments. Our results indicate that commensal gut bacteria are involved in physiological resistance to insecticide and support a role for both fat body and gut microbial communities in the regulation of reproductive life history traits that may contribute to the establishment of resistance at the population level.

## Materials & methods

### Cockroach strains and maintenance

Three strains of German cockroach (*Blatella germanica*) were used in our experiments. The susceptible Orlando normal strain (ORL), which was colonized prior to the widespread use of insecticides (>60 years ago), was maintained without insecticide selection [30]. Further, an indoxacarb-resistant field strain (DE) was collected from Destin, FL in 2011 and subsequently maintained in the laboratory without insecticide selection. A portion of this field-collected colony was also separated and maintained under indoxacarb bait selection pressure by periodic treatment with Advion cockroach gel bait containing 0.6% indoxacarb (Syngenta, Basel, Switzerland), as previously described [9]. This strain was termed DEA. All cockroaches were reared in 15 x 9 inch plastic arenas held in environmentally controlled rooms at 27 C and 45% relative humidity on a 12:12 light:dark cycle. Insect colonies and experimental insects were provided with cardboard harborages, water, and dog chow (Purina, St. Louis, MO) unless otherwise indicated.

### Antibiotic and insecticide treatments

**Fig 1:**
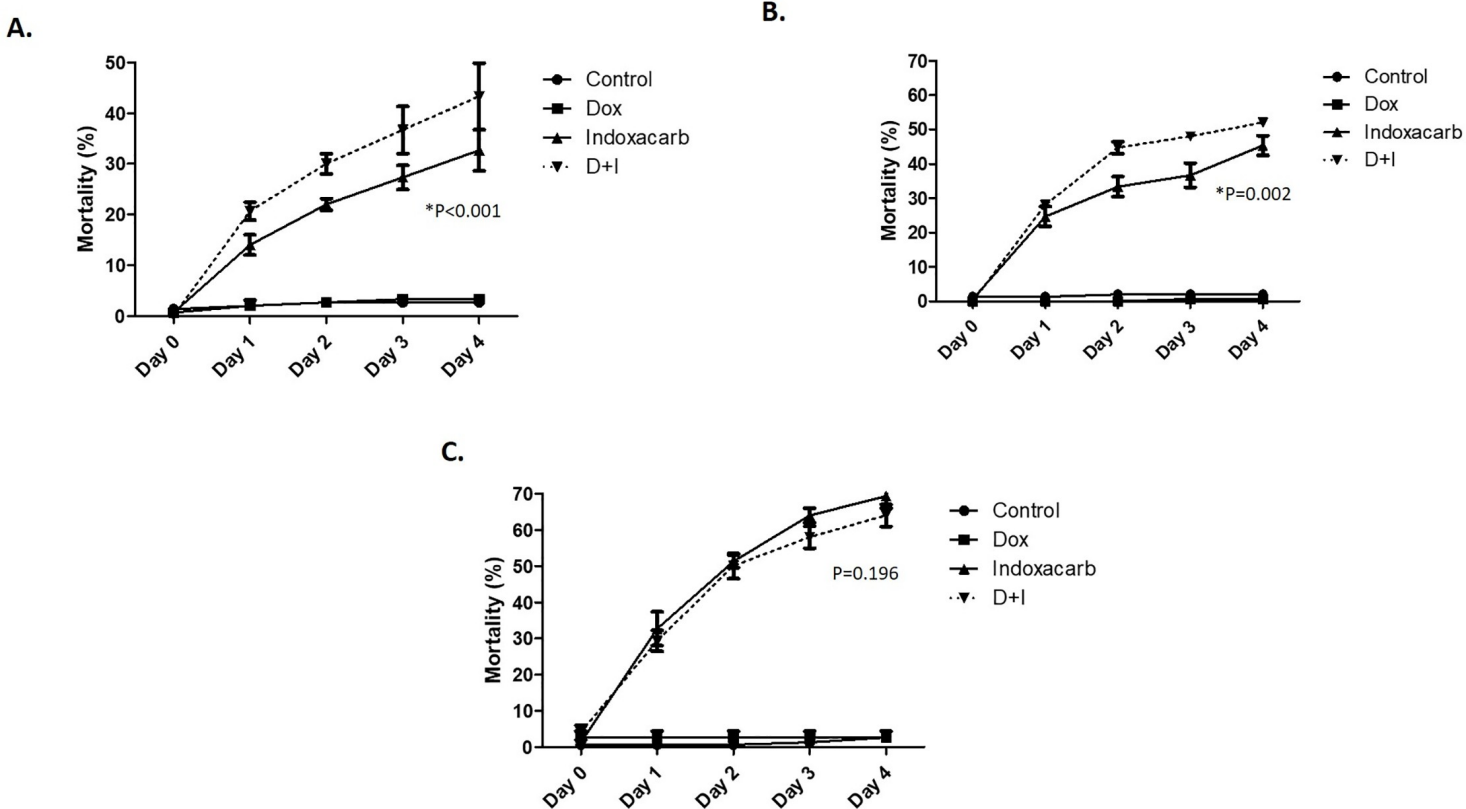
Doxycycline increases oral insecticide toxicity in resistant cockroaches. **(A)** Resistant, bait-selected DEA cockroaches, **(B)** Resistant DE cockroaches, and **(C)** susceptible ORL cockroaches were exposed to customized gel baits containing 0.05% indoxacarb, 0.5% doxycycline, or a combination of both, and mortality was assessed over a period of 4 days. N = 3 replicates of 50 insects.

**Fig 2:**
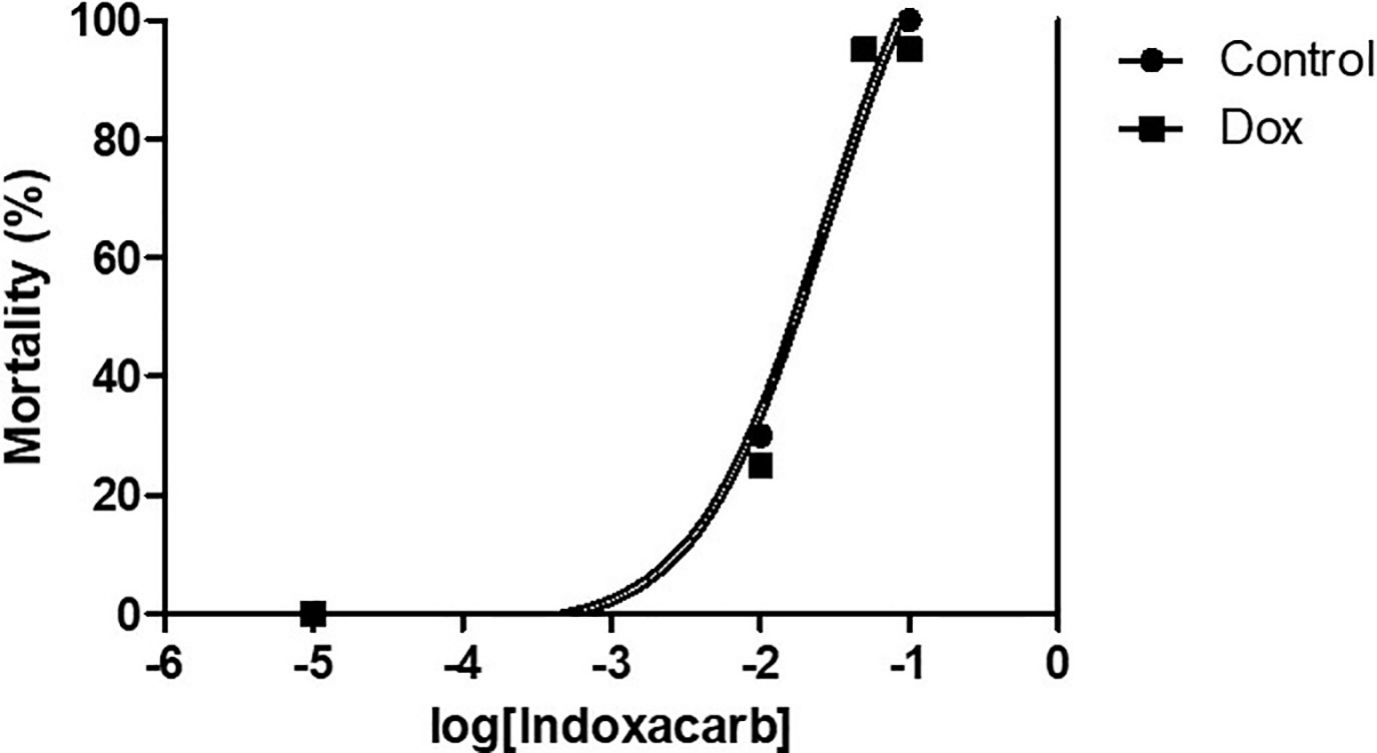
Doxycycline does not affect topical insecticide toxicity. Male cockroaches of the resistant, bait-selected DEA strain were topically treated with various doses of indoxacarb in acetone and mortality was assessed over a 24-hour period. The LD_50_ was determined by nonlinear regression analysis in cockroaches treated with insecticide only (circles) and cockroaches pre-treated with 0.5% doxycycline for 7 days prior to insecticide treatment (squares). N = 20 insects per treatment.

For analyses of the effects of bacteria on insecticide resistance **(Figs 1 and 2)**, the antibiotic doxycycline (Sigma Aldrich, St. Louis, MO) was provided in either a customized gel bait or in water, while indoxacarb was administered either in the same gel or topically for determination of the LD_50_. Gel baits for oral toxicity experiments **(Fig 1)** consisted of the following by weight: 48% ground chicken feed (Purina), 1% potassium sorbate, 0.5% agar, and either 0.5% doxycycline, 0.05% indoxacarb, or both, with the balance consisting of distilled water. Active ingredient doses were selected to both minimize effects on gel palatability and allow for maximum differentiation of mortality between resistant and susceptible insect strains. Baits were administered to experimental replicates of 50 healthy cockroaches (15 males, 10 females, 25 nymphs/group) in plastic arenas under standard conditions and mortality was assessed over a period of 4 days. Mortality curves were compared by two-way ANOVA using GraphPad Prism 5 (Graphpad Software Inc., La Jolla, CA).

For determination of the LD_50_ of topically applied indoxacarb **(Fig 2)**, healthy male cockroaches from the resistant DEA colony were pre-treated by adding 0.5% doxycycline to their water source for 7 days. Insects were then anesthetized on ice prior to applying 1 μL of acetone with either no insecticide (control), or one of a series of concentrations of indoxacarb (0.1μg-0.001μg/μL) that were chosen such that at least 3 concentrations would yield 1–99% mortality [9]. Applications were made to the ventral thorax between the coxae of 20 cockroaches for each concentration using a microapplicator (Hamilton, Reno, NV) equipped with a glass syringe. After application, insects were transferred into arenas and maintained under standard conditions. Mortality was examined after 24 hours and moribund cockroaches (those unable to right themselves when on their back) were counted as dead in our analyses. The LD_50_ was determined by nonlinear regression analysis using GraphPad Prism 5.

### Microbiota sequencing

**Fig 3:**
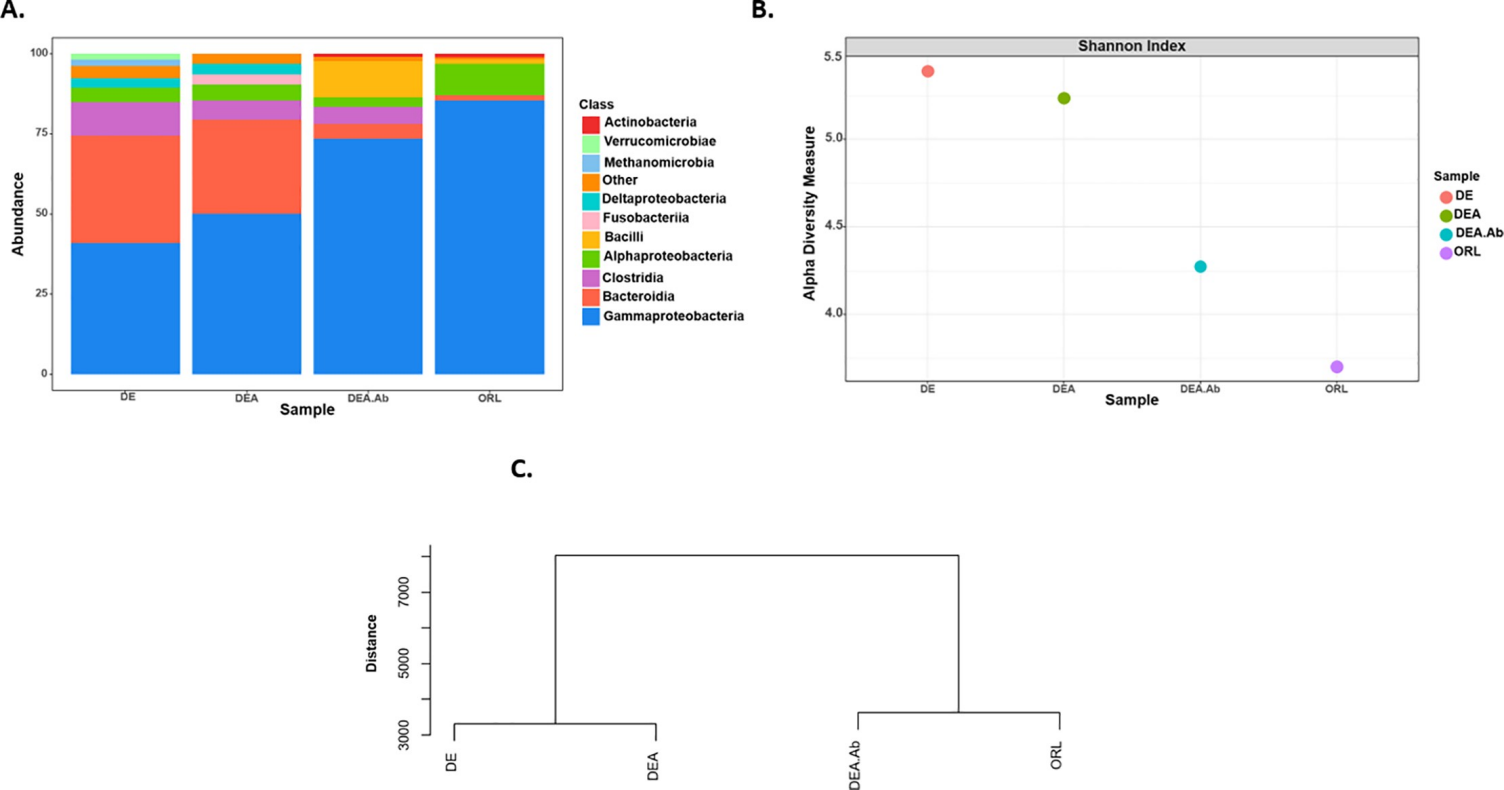
The gut microbiota varies among resistant and susceptible German cockroach strains. Whole guts from untreated cockroaches, or cockroaches continuously exposed to 0.5% doxycycline for 4 days were dissected and DNA was isolated for PCR amplification and sequencing of bacterial 16S rRNA genes. **(A)** Relative abundance of ASVs that were called to the taxonomic rank of Class. Taxa with <1% relative abundance were grouped together as “other.” **(B)** Alpha diversity Index (Shannon Index) of amplicon sequence variants (ASVs). **(C)** Hierarchical clustering analysis (beta diversity) of ASVs.

Sequencing of the cockroach microbiota **(Fig 3)** was carried out at Molecular Research DNA Lab (Shallowater, TX, USA) using an Illumina MiSeq system (Illumina, San Diego, CA) according to the manufacturer’s guidelines. For each strain or treatment group, whole guts (foregut, midgut, hindgut) were dissected from 6 male cockroaches using sterilized tools. Insects were starved of food for 24 hours to minimize non-stably associated bacteria and were rinsed with ethanol and sterile water immediately before dissection. Gut tissues from the 6 insects were pooled together to account for variation between individuals from the same treatment, though this is expected to be minimal given cockroach aggregation behavior [31]. DNA was extracted using the DNeasy Powersoil Kit (Qiagen, Hilden, Germany). Primers for the V4 variable region of the bacterial 16S rRNA gene with barcode on the forward primer [32] were used to conduct PCR using the HotStarTaq Plus Master Mix Kit (Qiagen). Cycle conditions were as follows: 94°C for 3 minutes, followed by 28 cycles of 94°C for 30 seconds, 53°C for 40 seconds and 72°C for 1 minute, after which a final elongation step at 72°C for 5 minutes was performed. Following PCR, products were checked in 2% agarose gels to verify successful amplification. The different samples were then pooled together in equal proportions based on their molecular weight and DNA concentrations. Pooled samples were purified using calibrated Ampure XP beads (Beckman Coulter, Brea, CA) and subsequently used to prepare a DNA library through the Illumina TruSeq DNA library preparation protocol.

### Analysis of sequencing data

16S rRNA sequencing reads were demultiplexed using MRDNA software (http://www.mrdnafreesoftware.com). Reads were then processed and assembled into amplicon sequence variants (ASV) using the most recent release of dada2 in R (https://benjjneb.github.io/dada2/index.html) [33]. Forward reads shorter than 240 base pairs and reverse reads shorter than 160 base pairs were discarded, as well as chimeric reads and any reads with more than 2 expected errors (see **S1 Table** for raw sequence statistics). Taxonomy was assigned using the dada2 formatted greengenes 13.8 training dataset (https://zenodo.org/record/158955#.W61TS2hKist) [34] and rarefaction analysis was done to confirm that sequencing captured all ASVs. Contaminating reads from the *Blattabacterium* genus, which were present due to small amounts of difficult to remove fat body tissue surrounding the dissected guts, were subsequently removed using the prune taxa function in phyloseq. Relative abundance plots were constructed using phyloseq and ggplot2 [35] and Pearson’s chi-square testing was conducted to identify significant differences in distribution between samples at the class level. Shannon Index analysis of alpha diversity was performed using phyloseq and beta diversity analysis was performed using a complete-linkage hierarchical clustering model with the base R stats package, with Euclidean distance used as the distance metric for the dendrogram. Venn diagrams were generated using ggforce [36]. All raw sequencing reads associated with the manuscript were deposited into the NCBI Sequence Read Archive (SRA) under accession number SRP145206, BioProject: PRJNA470750

### Fecal transplant

**Fig 4:**
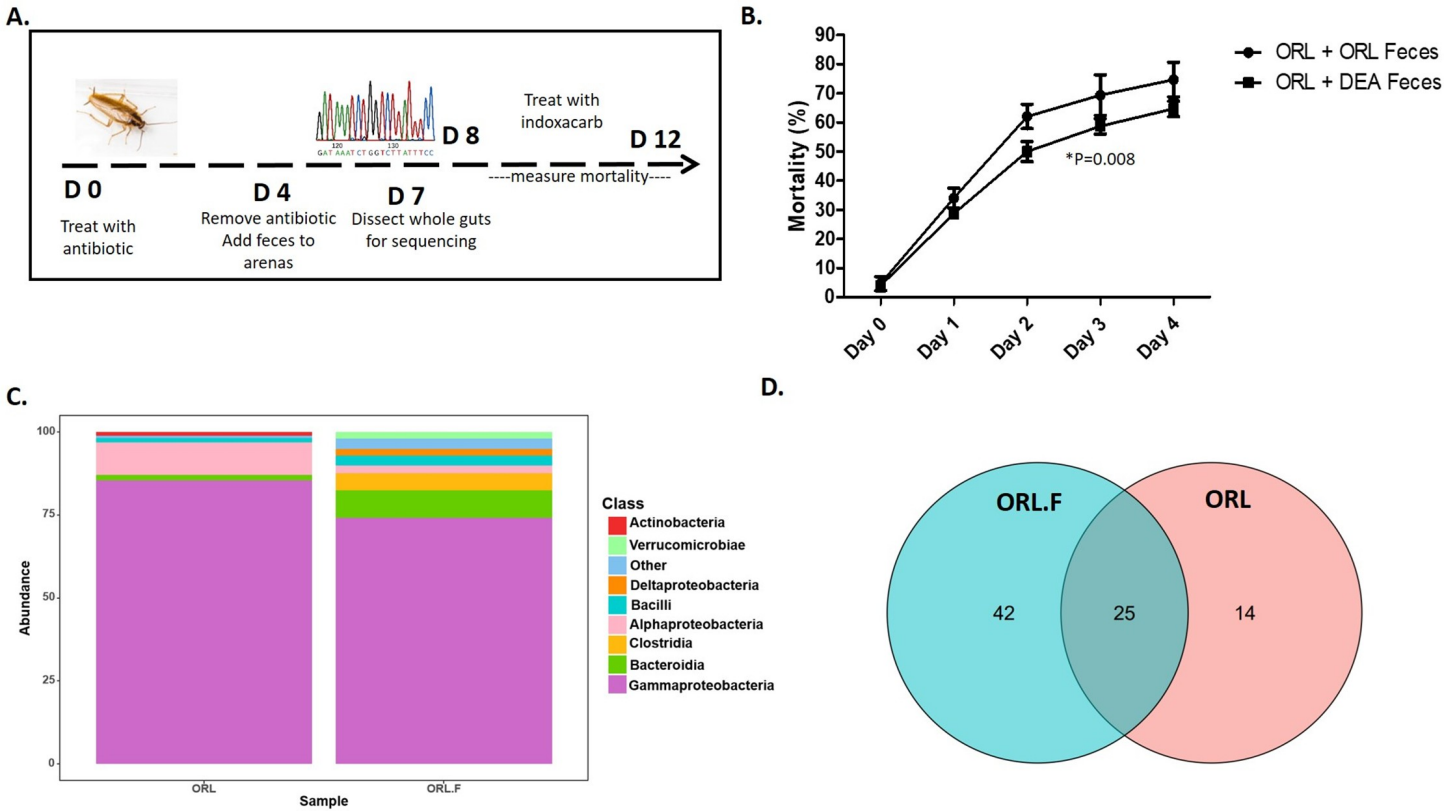
Transplant of gut bacteria from resistant insects partially recapitulates resistance in susceptible cockroaches. **(A)** Schematic of experimental timeline for fecal transplant. **(B)** Susceptible cockroaches (ORL) transplanted with feces from a resistant, bait-selected colony (DEA) were exposed to baits containing 0.05% indoxacarb. Mortality was assessed over a period of 4 days and compared to cockroaches receiving a control transplant from their own susceptible colony. N = 3 replicates of 50 insects. **(C)** The relative abundance of bacterial ASVs that were called to the taxonomic rank of Class in susceptible cockroaches before (ORL) and after (ORL.F) fecal transplant from the resistant strain. Taxa with <1% relative abundance were grouped together as “other.” **(D)** Venn diagram of assigned taxa in susceptible cockroaches before (ORL) and after (ORL.F) fecal transplant from the resistant strain.

For fecal transplant experiments **(Fig 4)**, replicates of 50 insects of the susceptible lab strain (15 males, 10 females, 25 nymphs) were placed into arenas and pre-treated with 0.5% doxycycline by addition to their water source for 4 days. After 4 days, the antibiotic was removed and each arena received 1 gram of feces from a resistant, bait-selected colony, or from a susceptible colony (control). For the following 3 days, cockroaches were starved of food to promote the consumption of feces. Afterwards, dog chow was added to the arenas and cockroaches were allowed to recover from starvation for 24 hours before insecticide exposure. Insecticide treatments were carried out using customized gel baits as described above and survival curves were compared by two-way ANOVA using Graphpad Prism 5.

### Incorporation of synergists into indoxacarb and antibiotic gel baits

**Fig 5:**
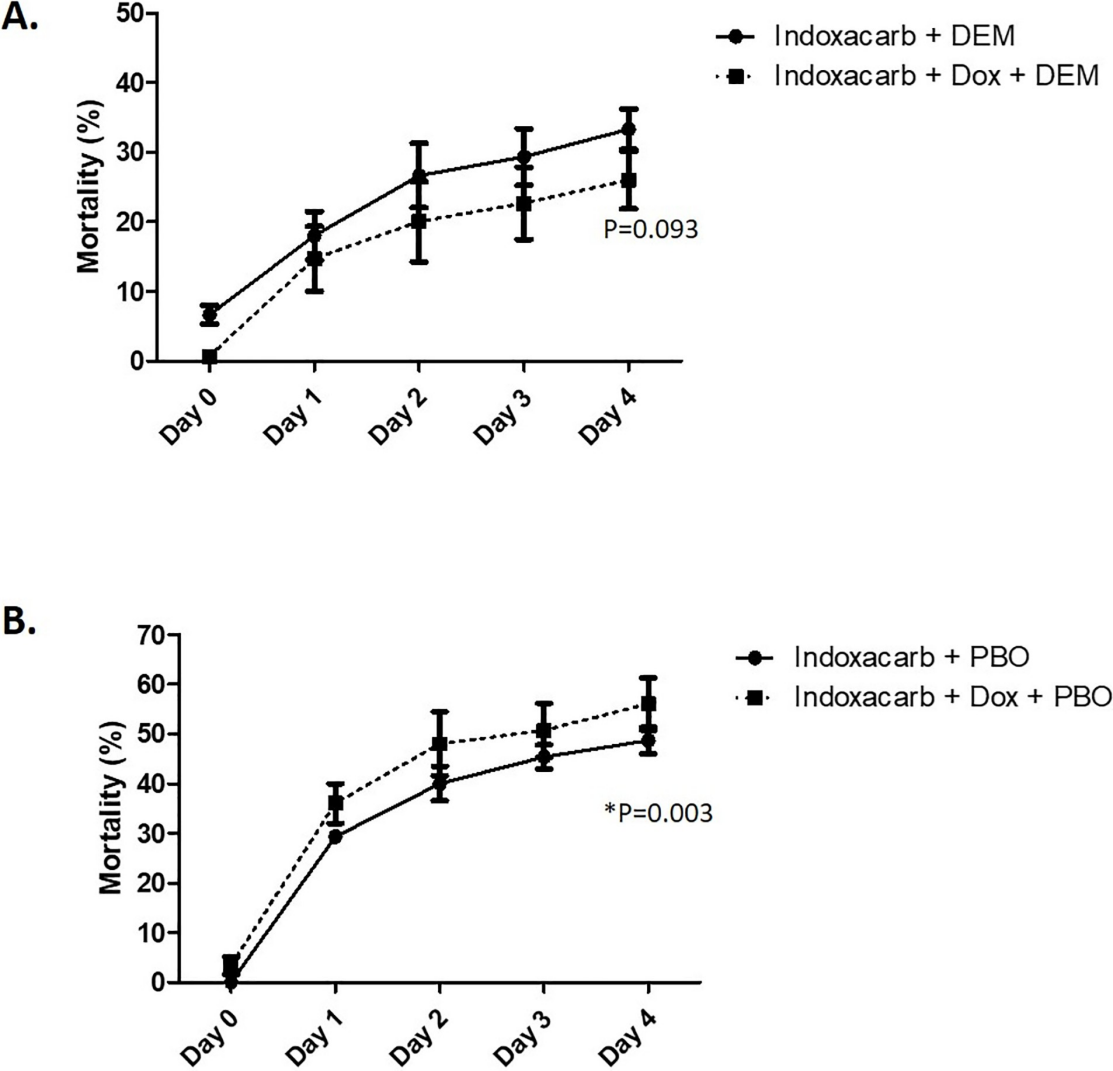
Inhibition of glutathione-S-transferase prevents the effect of doxycycline on indoxacarb toxicity. Resistant, bait-selected cockroaches (DEA) were exposed to customized gel baits containing indoxacarb (0.05%) +/- doxycycline (0.5%) and either **(A)** the glutathione-S-transferase inhibitor DEM, or **(B)** the cytochrome P450 monooxygenase inhibitor PBO. Mortality was assessed over a period of 4 days. N = 3 replicates of 50 insects.

The insecticide synergists piperonyl butoxide (PBO), a cytochrome P450 monooxygenase inhibitor, and diethyl maleate (DEM), a glutathione-S-transferase inhibitor, were incorporated into customized gel baits at a final concentration of 0.25% using the aforementioned formulas containing indoxacarb and doxycycline **(Fig 5)**. Cockroaches were treated with these baits and mortality over a period of 4 days was recorded. Survival curves were analyzed by two-way ANOVA using GraphPad Prism 5.

### Life history analysis

**Fig 6:**
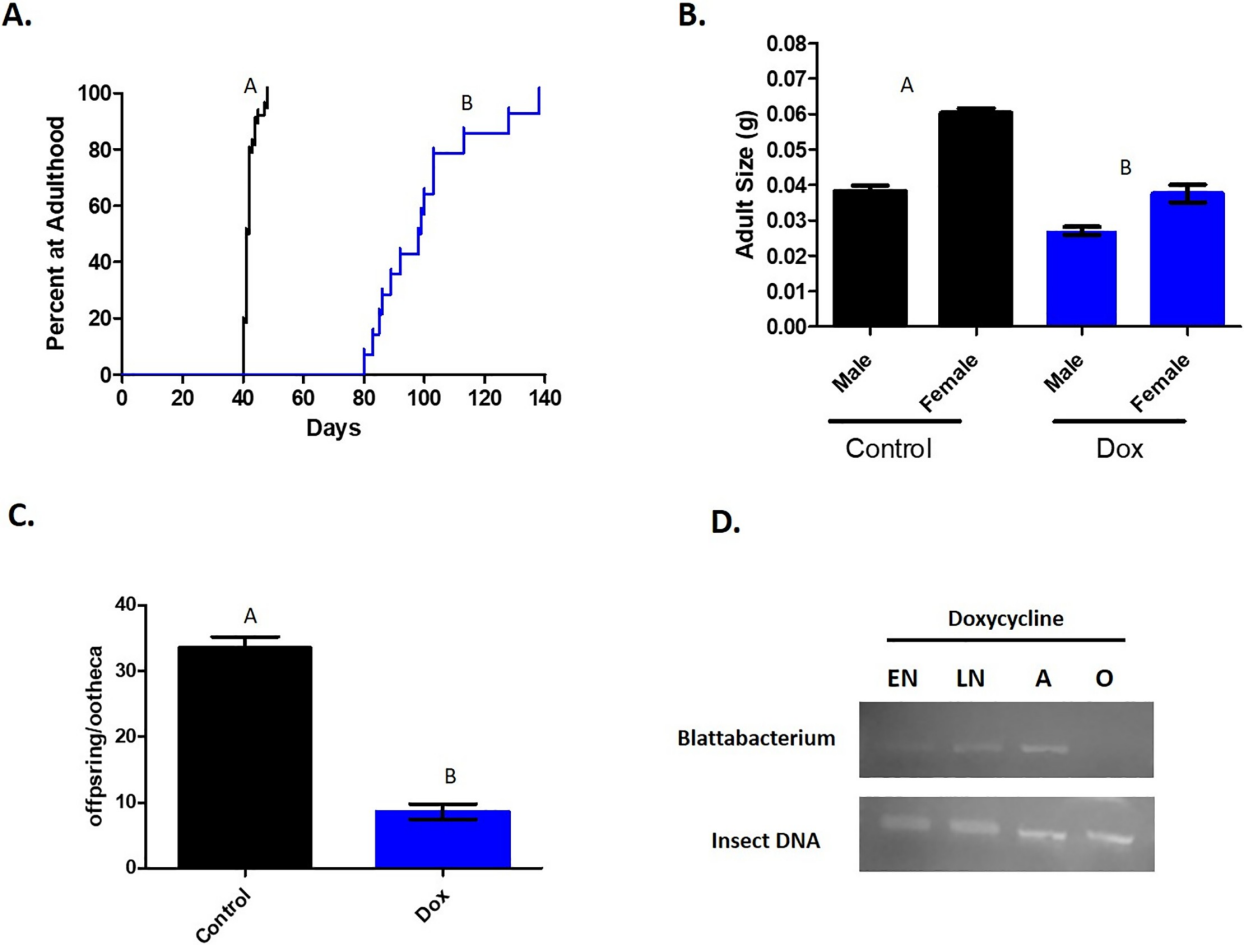
Disruption of the microbiota reduces the reproductive fitness of German cockroaches. **(A)** Time to adulthood (n = 14–38), **(B)** adult body size (n = 20), and **(C)** fecundity (n = 5–18) were measured in untreated cockroaches (black) and cockroaches continuously exposed to 0.5% doxycycline (blue). **(D)** *Blattabacterium* was detected by PCR in early nymphs (EN), late nymphs (LN), and adults (A) that were continuously exposed to doxycycline, but not in the offspring (O) of these cockroaches. Different letters within graphs indicate statistically significant differences between groups.

Analyses of the effects of microbes on life history traits were performed on the susceptible ORL strain **(Fig 6)**. In control groups, individual oothecae (egg cases) were collected prior to hatching and placed into experimental areas under the standard conditions described above. Once the oothecae hatched, cockroaches were monitored at regular intervals and the time taken to reach adulthood was recorded. Cockroaches were also weighed at adulthood. After these measurements were taken, the insects were maintained in the same experimental containers until females developed mature oothecae. These oothecae were then collected, placed into new arenas individually, and the number of viable offspring produced from each ootheca was recorded. For comparison, the same measurements were taken from cockroaches that were continuously exposed to 0.5% doxycycline (Sigma Aldrich) by addition to their water source upon hatching. Development curves were analyzed by Mantel-Cox Log rank test, while adult weight and fecundity data were analyzed by t-test using Graphpad Prism 5.

### PCR

The presence of *Blattabacterium* throughout development in antibiotic-treated cockroaches was examined by PCR **(Fig 6)**. Single cockroaches were collected into Extract-N-Amp tissue preparation buffer (Sigma Aldrich) and homogenized using a pestle. DNA extraction was performed according to the manufacturer’s protocol and 4 μL of extraction solution was used for amplification of insect or *Blattabacterium* sequences with Extract-N-Amp PCR ReadyMix on a MiniPCR system (Amplyus, Cambridge, MA). The primers and cycle conditions used were as previously described to amplify a 460 base pair fragment of the 16S RNA gene of *B*. *cuenoti* [37–38] or a 400 base pair fragment of insect mitochondrial DNA [39]. Amplified PCR products were verified by electrophoresis on 1% agarose gels using a bluegel integrated electrophoresis and visualization system (Amplyus). The experiment was replicated with 2 independent sets of cockroaches. Original images of gels used in the figure are presented in the supplementary material.

## Results

### Antibiotic treatment enhances oral but not topical toxicity of indoxacarb

We began our investigation of microbe-mediated insecticide resistance in the German cockroach by examining the effects of antibiotic treatment on insecticide toxicity. Specifically, we first determined the outcome of combining doxycycline (0.5%) and the commonly used oxadiazine insecticide, indoxacarb (0.05%), in gel baits fed to 3 cockroach strains with differential levels of resistance **(Fig 1)**. In these experiments, neither blank gels containing only chicken feed nor gels containing doxycycline without indoxacarb caused any mortality, while gels containing indoxacarb alone led to variable mortality that corresponded with the resistance status of the strain being tested. More importantly, in the resistant, bait-selected DEA (**Fig 1A**, ANOVA, n = 3, P<0.001) and resistant DE (**Fig 1B**, ANOVA, n = 3, P = 0.002) strains, gels containing a combination of doxycycline and indoxacarb elicited significantly higher mortality than the gels containing the insecticide alone. This was the case despite the addition of antibiotic slightly diminishing palatability and resulting in less bait consumption by the insects **(S1 Fig)**.

Unexpectedly, we did not observe the same mortality trend in the susceptible laboratory strain. In these cockroaches, the effects of the combination gel were not statistically different from those of the indoxacarb gel (**Fig 1C**, ANOVA, n = 3, P = 0.196). These results indicated that the impact of commensal microbes on insecticide resistance in the German cockroach is strain-dependent and not conserved across populations. One plausible explanation for the observed specificity is that microbe-mediated resistance is derived from microbial populations in the gut that may vary among cockroach strains. To test this hypothesis, we then examined the effect of antibiotic treatment on the toxicity of indoxacarb topically applied directly to the cuticle **(Fig 2)**. This approach bypassed the gut barrier that is encountered by insecticides delivered in bait and prevented interaction between indoxacarb and the gut microbiota. Doxycycline had no effect on indoxacarb toxicity when it was administered in this fashion. That is, in the resistant, bait-selected strain, topically applied indoxacarb was equally toxic to cockroaches that were pre-treated with doxycycline for seven days and those that received no antibiotic treatment (LD_50_ of 0.0276 μg/insect and 0.0263 μg/insect, respectively). These results were in direct contrast to data obtained in our oral toxicity experiments and further suggested that the impact of doxycycline on the oral toxicity of indoxacarb is dependent on antibiotic effects against microbial populations in the cockroach gut.

### The gut microbiota differs among resistant and susceptible cockroach strains

To identify potential disparities among resistant and susceptible cockroaches that could explain the variable effect of doxycycline on indoxacarb toxicity, we next sequenced the gut microbiota of each strain under basal conditions, as well as after antibiotic treatment in the bait selected, resistant strain. Consistent with previous analyses [20–21, 23,25], albeit with some deviation, the microbiota of our insects consisted primarily of Proteobacteria, Bacteroidia, Firmicutes (i.e. Bacilli, Clostridia) and Fusobacteria. The relative abundances of these taxa in the gut of German cockroaches can vary drastically among individuals from different locations, and even among individuals in a laboratory environment kept under different dietary regimes [21,40]. In our samples, Gammaproteobacteria were prevalent due to the presence of what may be a non-*Blattabacterium* endosymbiont. Nonetheless, several differences in the remaining taxa were also observed between strains and treatment groups in our studies **(Fig 3A)**. Primarily, we found that alpha diversity was greater in resistant cockroaches than in the susceptible lab strain **(Fig 3B)** and these strains (DEA vs ORL) harbored a significantly different distribution of microbial taxa (chi-square, P<0.001). Treatment with antibiotic (DEA vs DEA.Ab) reduced alpha diversity and significantly altered the distribution of microbial taxa (chi-square, P<0.001), while selection with insecticidal bait (DE vs DEA) appeared to have minimal impact on diversity but still significantly altered the distribution of the taxa present (chi-square, P<0.001). Only the resistant but not insecticide selected strain (DE) harbored Methanomicrobia and Verrucomicrobiae at an abundance >1%, while the bait-selected strain (DEA) exclusively harbored Fusobacteria at an abundance >1%, but only in the absence of antibiotic treatment. Clostridia and Deltaproteobacteria were noticeably lacking in susceptible cockroaches, as neither was present at a relative abundance >1%. Additionally, the relative abundance of Bacteroidia was reduced in the susceptible strain. Treatment of the bait-selected, resistant strain (DEA) with doxycycline reduced the abundance of Deltaproteobacteria and Bacteroidia. In total, our beta diversity analysis showed that the communities of the resistant strains before and after bait selection were most similar and markedly dissimilar from the susceptible strain, but treatment of the bait-selected strain (DEA) with doxycycline shifted the composition of the microbiota towards one that more closely resembled that of the susceptible strain **(Fig 3C)**. The aforementioned patterns were apparent even when highly abundant Gammaproteobacteria were not considered **(S2 Fig)**.

### Fecal transplant increases insecticide resistance in susceptible cockroaches

Having implicated variation in the gut microbiota as a potential contributor to insecticide resistance, we sought to determine whether resistance could be recapitulated by transfer of microbes from the gut of the resistant, bait-selected strain to the susceptible strain **(Fig 4A)**. We hypothesized that this transfer could be accomplished by supplementing cockroaches with conspecific feces. Indeed, when susceptible cockroaches were fed feces obtained from a colony of resistant counterparts prior to insecticide treatment, mortality after 4 days of exposure to a gel bait containing indoxacarb was decreased relative to groups fed feces from their own susceptible colony as a control (**Fig 4B**, ANOVA, n = 3, P = 0.008). Sequencing of the gut microbiota of susceptible roaches transplanted with feces from the resistant, bait-selected strain further revealed that fecal transplant significantly altered the distribution of microbial taxa (chi-square, P<0.001) and introduced a number of unique assigned taxa that were not previously present **(Fig 4C and 4D)**. In particular, the abundance of Bacteroidia appeared to expand, while Clostridia and Deltaproteobacteria, which are present in the resistant strains but negligible in the susceptible strain, were detected at augmented levels. Therefore, microbiota-mediated resistance can be partially recapitulated in susceptible cockroaches through a transfer of gut microbes that occurs during coprophagy.

### Inhibition of glutathione-S-transferase prevents the effect of doxycycline on indoxacarb toxicity

Experiments involving the incubation of indoxacarb in bacterial cultures derived from cockroach feces produced no evidence of direct detoxification by these microbes **(S3 Fig).** We instead hypothesized that the gut microbiota may confer insecticide resistance through indirect detoxification, by enhancing the expression or activity of endogenous host detoxification pathways. This hypothesis was tested by inhibiting several key enzymes involved in xenobiotic metabolism. When we incorporated the glutathione-S-transferase (GST) inhibitor diethyl maleate (DEM) into gel baits containing indoxacarb, mortality in resistant, bait-selected cockroaches (DEA) exposed to the baits was equal regardless of whether or not the bait contained doxycycline **(Fig 5A**, ANOVA, n = 3, P = 0.093). These results were inconsistent with those obtained in the same strain without GST inhibition **(Fig 1)**. In other words, inhibition of GST prevented doxycycline-mediated enhancement of indoxacarb toxicity against resistant cockroaches. However, in the context of cytochrome P450 inhibition using PBO, the effects of doxycycline were conserved, as addition of antibiotic to indoxacarb gel baits increased toxicity in the resistant, bait-selected strain relative to baits containing indoxacarb alone **(Fig 5B**, ANOVA, n = 3, P = 0.003). In contrast to GST inhibition, these results were consistent with the effects of doxycycline on indoxacarb toxicity in the same cockroaches not treated with inhibitors.

### Perturbation of the microbiota adversely affects reproductive fitness

Insecticide resistance is propagated through a cockroach population because individuals that are able to survive insecticide exposure gain a reproductive advantage over susceptible insects in an environment where insecticide is present. At the same time, when the pressure of insecticide selection is removed, resistance is often accompanied by one or more fitness costs. Thus, our study also examined several life history parameters as a readout for the reproductive fitness of antibiotic-treated cockroaches, to determine if the microbiota is also involved in this evolutionary aspect of resistance **(Fig 6).** From the time of hatching from the ootheca, control cockroaches took an average of 41.5 days to reach adulthood **(Fig 6A)**. Meanwhile, cockroaches continuously exposed to doxycycline took an average of 98.5 days, a statistically significant increase of more than 2-fold (Mantel-Cox Log rank, P<0.0001). When adults from each treatment group were weighed, additional differences were apparent **(Fig 6B)**. Both males (0.038g vs 0.027g/insect) and females (0.06g vs 0.038g/insect) treated with antibiotic were significantly smaller in size when compared to control cockroaches (t-test, P<0.05). Antibiotic treatment further led to a marked drop in fecundity relative to controls **(Fig 6C**, 33.5 vs 8.6 viable eggs/ootheca, t-test, P<0.05). Interestingly, while short-term doxycycline treatment had an effect on the gut microbiota **(Fig 3)**, it did not rapidly remove *Blattabacterium*. Rather, *Blattabacterium* was detectable by PCR throughout the lifespan of cockroaches treated with antibiotic. Only during oogenesis did *Blattabacterium* appear to be fully eliminated, as it was not present in the offspring of antibiotic-treated cockroaches **(Fig 6D and S4 Fig)**. Taken together these results indicate that the microbial community in the gut is not only directly involved in the physiological response to insecticide, but also contributes to reproductive fitness, likely in combination with *Blattabacterium*-mediated effects.

## Discussion

In the present study, we reveal that the microbiota of the German cockroach contributes to resistance to an oxadiazine sodium channel blocker, as well as multiple aspects of the insect’s life history. These results significantly advance current knowledge of the cockroach microbiota and the development of insecticide resistance from the biochemical to the evolutionary level.

At the core of our findings are two key observations: (1) microbial regulation of resistance to indoxacarb is specific to select, field-derived laboratory cockroach colonies, and (2) microbial regulation of resistance applies only to orally administered insecticide. These two stipulations indicate that resistance is not mediated by the fat body endosymbiont, *Blattabacterium*, which is present in all German cockroach strains and does not localize to the gut [41]. They also argue against the involvement of off-target antibiotic effects on host physiology. Instead, the ability of antibiotic treatment to increase the toxicity of indoxacarb in certain cockroach strains appears to be due to its effects on bacteria that are present in the gut of resistant, but not susceptible cockroaches. Sequencing of the gut microbiota of susceptible and resistant strains indeed revealed key differences between the two communities. Notably, the diversity of the microbiota was substantially lower in the susceptible Orlando strain that was colonized >60 years ago, suggesting that some microbial species and their respective functions may have been lost as part of long-term laboratory adaptation process. A specific, causative agent of resistance was not pin-pointed, but across our experiments there appeared to be a correlation with the presence of Deltaproteobacteria and Clostridia [42], which are known to be involved in the response to xenobiotics in the mammalian gut. Alternatively, it may be that resistance is not caused by the presence of a singular taxa or species, but rather depends on the overall diversity and/or composition of the microbiota and its metabolic dynamics that are disrupted during dysbiosis caused by antibiotic treatment [43]. Testing for microbe-mediated resistance using antibiotics with varied spectra of activity in both indoxacarb resistant strains and strains that are resistant to other insecticides may help determine the prevalence of this phenomenon as well as the bacteria responsible.

As it stands, the mechanism for microbe-mediated resistance to indoxacarb in the German cockroach has not been fully elucidated. It is possible that exposure to the insecticide may select for insects that harbor gut microbes that contribute to survival under this pressure. Similar shifts in the composition of the gut microbiota have been reported in aphids following treatment with spirotetramat [44]. Our fecal transplant experiments demonstrate that shifts in the microbial community of the gut, along with associated resistance traits, can be passed between individuals through coprophagy. These results support recent work evidencing vertical transmission of the microbiota via coprophagy in *B*. *germanica* [23, 40], and expand the significance of this behavior to include a putative role in the propagation of insecticide resistance. Intriguingly, we found no evidence of direct detoxification of indoxacarb by bacteria cultured from the feces of resistant cockroaches. It should be noted, however, that these assays only examined the effects of culturable bacteria in liquid LB media. Thus, the possibility remains that some microbes that are fastidious, anaerobic, or of low abundance contribute to direct detoxification of indoxacarb *in vivo* [45].

A more probable mechanism involves indirect detoxification of indoxacarb by cells of the gut (i.e. detoxification through endogenous host pathways induced by the microbiota). In the German cockroach, indoxacarb undergoes cytochrome P450-dependent biotransformation that influences its toxicity [46]. In our experiments, inhibition of this process using the synergist PBO did not affect microbe-mediated resistance. On the other hand, when GST-dependent metabolism was blocked using DEM, microbe-mediated resistance was reversed, suggesting that the microbiota boosts either GST gene expression or enzymatic activity, thereby contributing to detoxification. Indoxacarb resistance has been linked to GST activity in other insects, supporting this conclusion [47]. Moreover, in the mosquito *Anopheles stephensi*, symbiotic bacteria from the midgut can alter GST activity [48]. Therefore, the effects of the microbiota on GST in the cockroach gut should be explored further using molecular methods to determine if specific bacterial metabolites play a role in its function.

In addition to affecting insecticide resistance, we show that antibiotic treatment changes cockroach development and reproduction. However, because effects on life history are conserved in the susceptible cockroach strain, the microbes involved in this biology are almost certainly different from those that mediate insecticide resistance. Whether the effects of doxycycline on *Blattabacterium*, which may provide nutritional benefits to these causes [26,49], contribute to the phenotypes we observed is unclear given the unusual kinetics of *Blattabacterium* in response to antibiotic treatment. That is, while we and others [23] show that *Blattabacterium* endosymbionts do not appear to be killed by antibiotic exposure except during their transit to the developing oocyte, it is difficult to rule out sub-lethal antibiotic effects on these microbes that may impact their metabolic status. It is highly probable that the microbial community in the gut is involved in regulating metabolic rate, as in the American cockroach [50]. Because this community is efficiently targeted by doxycycline treatment, it is a more likely contributor to life history. Although the pathways by which the microbiota affects life history traits remain unknown, our studies lay a foundation for future work in this area. Of additional interest is the potential involvement of endosymbionts besides *Blattabacterium*, such as *Wolbachia*, which have been detected in a small percentage of German cockroach populations but remain understudied in this insect [51]. Regardless of the particular microbes involved, the above findings have important implications. By affecting reproduction and development, the microbiota could influence the growth of resistant populations under insecticide selection pressure and also explain the fitness costs commonly associated with resistance outside of this context [52]. That is, under exposure to insecticide, the microbial community in the gut may be altered. At the individual level, we show that these shifts can contribute to physiological resistance. Simultaneously, alterations in the microbiota may, depending on environmental pressure, increase or decrease reproductive fitness leading to changes at the population level.

The relevance of our results to the control of resistant cockroach infestations outside the laboratory ultimately remains to be determined, as the impact of microbe-mediated resistance is likely to be dependent on interactions with other more prevalent host mechanisms of resistance. Moreover, the microbiome of field-collected cockroaches was found to be more variable than that of insects raised in the lab [40], and this variation may affect microbe-mediated resistance. No less, if the phenomenon presented here is confirmed in field populations, the approach of targeting commensal bacteria shows strong promise as a tool for integration into cockroach IPM programs. While our results suggest that this strategy is unlikely to be effective in boosting the toxicity of contact insecticides, antibacterial compounds can be easily incorporated into readily consumed bait products and their delivery through this route would require minimal effort but could substantially improve control with ingested active ingredients. Notably, a two-pronged approach that targets resistant populations by simultaneously enhancing insecticide efficacy and stunting the growth of insects that survive insecticide exposure represents a novel strategy for cockroach management.

## Supporting information

S1 Fig Doxycycline reduces consumption of gel baits by cockroaches. Customized gel baits containing indoxacarb alone (0.05%) or in combination with doxycycline (0.5%) were placed side-by-side in experimental arenas and consumption over a 4-hour period was determined by weight. The experiment was independently replicated 3 times using arenas with varied cockroach densities.
(TIF)

S2 Fig Gut microbiota composition excluding Gammaproteobacteria. Reads assigned to the Class Gammaproteobacteria were excluded from analysis and the relative abundance of the remaining taxa was determined.
(TIF)

S3 Fig Incubation with fecal bacteria does not reduce the toxicity of indoxacarb. Cultures of *E*. *coli* (control) or fecal bacteria from the bait-selected, resistant strain (DEA) were grown overnight in liquid LB containing 1 mg/ml indoxacarb and used in place of water to formulate customized gel baits with a final indoxacarb concentration of 0.05%. Susceptible cockroaches (ORL) were exposed to baits and mortality was assessed over a period of 4 days. N = 3 replicates of 50 insects.
(TIF)

S4 Fig Original images of gels used in Fig 6 of the manuscript. (TIF)

S1 Table Raw sequence statistics. (DOCX)

S1 File Supplementary materials & methods. (DOCX)

## References

[1] Garcia F., Notario MJ., Cabanas JM., Jordano R., Medina LM. Incidence of Bacteria of Public Health Interest Carried by Cockroaches in Different Food-Related Environments. J Med Entomol. 2012, 49: 1481–1484. 23270179 10.1603/me12007

[2] Menasria T., Moussa F., El-Hamza S., Tine S., Megri R., Chenchouni H. Bacterial load of German cockroach (Blattela Germanica) found in hospital environment. Pathog Glob Health. 2014, 108: 141–147. 10.1179/2047773214Y.0000000136 24766338 10.1179/2047773214Y.0000000136 PMC4083176

[3] Pai HH., Chen WC., Peng CF. Isolation of bacteria with antibiotic resistance from household cockroaches (*Periplaneta americana* and *Blattella germanica*). Acta Tropica. 2005, 93: 259–265. 10.1016/j.actatropica.2004.11.006 15716054 10.1016/j.actatropica.2004.11.006

[4] Fotedar R., Banerjee U. Vector potential of the German cockroach in dissemination of *Pseudomonas aeruginosa*. J Hospital Infection. 1993, 23: 55–59. 10.1016/0195-6701(93)90131-i 8095950

[5] Kopanic RJ., Sheldon BW., Wright CG. Cockroaches as Vectors of Salmonella: Laboratory and field trials. J Food Protection. 1994, 57: 125–135. 10.4315/0362-028X-57.2.125 31113148

[6] Rosenstreich DL, Eggleston P, Kattan M, Baker D, Slavin RG, Gergen P, et al The role of cockroach allergy and exposure to cockroach allergen in causing morbidity among inner-city children with asthma. N Engl J Med. 1997, 336: 1356–63. 10.1056/NEJM199705083361904 9134876 10.1056/NEJM199705083361904

[7] Do DC, Zhao Y, Gao P. Cockroach allergen exposure and risk of asthma. Allergy. 2016, 71: 463–74. 10.1111/all.12827 26706467 10.1111/all.12827 PMC4803579

[8] Cohn RD., Arbes SJ., Jaramillo R., Reid LH., Zeldin DC. National prevalence and exposure risk for cockroach allergen in U.S. households. Environ Health Perspect. 2006, 114: 522–526. 10.1289/ehp.8561 16581539 10.1289/ehp.8561 PMC1440774

[9] Liang D., Mcgill J., Pietri JE. Unidirectional cross-resistance in German cockroach (Blattodea: Blattellidae) populations under exposure to insecticidal baits. J Econ Entomol. 2017, 110: 1713–1718. 10.1093/jee/tox144 28541548 10.1093/jee/tox144

[10] Douglas AE. Multiorganismal insects: Diversity and function of resident microorganisms. Annu Rev Entomol. 2015, 60: 17–34. 10.1146/annurev-ento-010814-020822 25341109 10.1146/annurev-ento-010814-020822 PMC4465791

[11] Schlein Y. Lethal effect of tetracycline on tsetse flies following damage to bacterial symbionts. Experimentia. 1977, 33: 450–451. 10.1007/BF01922204 405237

[12] Sangare AK., Rolain JM., Gaudart J., Weber P., Raoult D. Synergistic activity of antibiotics combined with ivermectin to kill body lice. Int J Antimicrob Agent. 2016, 43: 217–223. 10.1016/j.ijantimicag.2016.01.001 26897755

[13] Kayya GP., Darji N., Otieno LH. Effects of bacteria, antibacterial compounds, and trypanosomes on tsetse reproduction and longevity. Int J Trop Insect Sci. 1987, 8: 271–220.

[14] Srivastava PN. & Auclair JL. Effects of antibiotics on feeding and development of the pea aphid *Acyrthosyphon pisum* (Harris) (Homeptera: Aphidae). Can J Zool. 1976, 54: 1025–1029.

[15] Bracke JW., Cruden DL., Markovetz AJ. Effect of metronidazole on the intestinal microflora of the American cockroach, *Periplaneta americana*. Antimicrob Agents Chemother. 1978, 13: 115–120. 626483 10.1128/aac.13.1.115 PMC352193

[16] Pietri JE., Liang D. The links between insect symbionts and insecticide resistance: Causal relationships and evolutionary tradeoffs. Ann Entomol Soc Am. 2018, 111: 92–97.

[17] Kikuchi Y. et al Symbiont-mediated insecticide resistance. Proc Natl Acad Sci USA. 2012, 109: 8618–8622. 10.1073/pnas.1200231109 22529384 10.1073/pnas.1200231109 PMC3365206

[18] Cheng D., Guo Z., Riegler M., Xi Z., Liang G., Xu Y. Gut symbiont enhances insecticide resistance in a significant pest, the oriental fruit fly *Bactrocera dorsalis*. Microbiome. 2017, 5: 13 10.1186/s40168-017-0236-z 28143582 10.1186/s40168-017-0236-z PMC5286733

[19] Sacchi L., Grigolo A., Laudani U., Ricevuti G., Dealessi F. Behavior of symbionts during oogenesis and early stages of development in the German cockroach, *Blatella germanica* (Blattodea). J Invert Pathol. 1985, 46: 139–152. 10.1016/0022-2011(85)90142-9 3930614

[20] Carrasco P., Perez-Cobas AE., van de Pol C., Baixeras J., Moya A., Latorre A. Succession of the gut microbiota in the cockroach *Blatella germanica*. Int Microbiol. 2014, 17: 99–109. 10.2436/20.1501.01.212 26418854 10.2436/20.1501.01.212

[21] Perez-Cobas AE., Maiques E., Angelova A., Carrasco P., Moya A., Latorre A. Diet shapes the gut microbiota of the omnivorous cockroach *Blatella germanica*. FEMS Microbiol Ecol. 2015, 91: doi: 10.1093 10.1093/femsec/fiv022 25764470

[22] Bertino-Gribaldi D., Medeiros MN., Vieira RP., Cardoso AM., Turque AS., Silveira CB., et al Bacterial community composition shifts in the gut of *Periplaneta americana* fed on different lignocellulosic materials. Springerplus. 2013, 2: 609 10.1186/2193-1801-2-609 24324923 10.1186/2193-1801-2-609 PMC3855920

[23] Rosas T., Garcia-Ferris C., Dominguez-Santos R., Llop P., Latorre A., Moya A. Rifampicin treatment *of Blatella germanica* evidences a fecal transmission route of their gut microbiota. FEMS Microbiol Ecol. 2018, 94: doi: 10.1093 10.1093/femsec/fiy002 29325007

[24] Gontang EA., Aylward FO., Carlos C., Glavina del Rio T., Chovatia M., Fern A., et al Major changes in microbial diversity and community composition across gut sections of a juvenile Panchlora cockroach. PLoS One. 2017, 12: e0177189 10.1371/journal.pone.0177189 28545131 10.1371/journal.pone.0177189 PMC5436645

[25] Zhang F., Sun XX., Zhang XC., Zhang S., Lu J., Xia YJ., Huang YH., Wang XJ. The interactions between gut microbiota and entomopathogenic fungi: A potential approach for biological control of *Blatella germanica* (L.). Pest Manage Sci. 2018, 74: 438–447. 10.1002/ps.4726 28888066

[26] Sabree ZL., Kambhampati S., Moran NA.Nutrient recycling and metabolic provisioning by *Blattabacterium*, the cockroach endosymbiont. Proc Natl Acad Sci USA. 2009, 106:19521–19526. 10.1073/pnas.0907504106 19880743 10.1073/pnas.0907504106 PMC2780778

[27] Wada-Katsumata A., Zurek L., Nalyanya G., Roelofs WL., Zhang, A., Schal, C. Gut bacteria mediate aggregation in the German cockroach. Proc Natl Acad Sci USA. 2015, 112:15678–15683. 10.1073/pnas.1504031112 26644557 10.1073/pnas.1504031112 PMC4697420

[28] Brooks MA., Richards AG. Intracellular symbiosis in cockroaches. I Production of aposymbiotic cockroaches. Biological Bulletin. 1955, 109: 22–39.

[29] Vicente CSL., Ozawa S., Hasegawa K. Composition of the cockroach gut microbiome in the presence of parasitic nematodes. Microbes Environ. 2016, 31: 314–320. 10.1264/jsme2.ME16088 27524304 10.1264/jsme2.ME16088 PMC5017809

[30] Silverman J., Ross MH. Behavioral resistance of field-collected German cockroaches (Blattodea: Blattellidae) to baits containing glucose. Environ Entomol. 1994, 23: 425–430.

[31] Berlanga M., Llorens C., Comas J., Guerrero R. Gut bacterial community of the Xylophagous cockroaches *Cryptocercus punctulatus* and *Parasphaeria boleiriana*. PLoS One. 2016, 10.1371/journal.pone.0152400 PMC4824515 27054320

[32] Caporaso JG., Lauber CL., Walters WA., Berg-Lyons D., Lozupone CA., Turnbaugh PJ. et al Global patterns of 16S rRNA diversity at a depth of millions of sequences per sample. Proc Natl Acad Sci USA. 2011, 15: 4516–4522. 10.1073/pnas.1000080107 PMC3063599 20534432

[33] R Core Team. R: A language and environment for statistical computing. 2017, R Foundation for Statistical Computing, Vienna, Austria. URL: https://www.R-project.org/.

[34] Callahan BJ., McMurdie PJ., Rosen MJ., Han AW., Johnson AJA., Holmes SP. DADA2: High resolution sample inference from Illumina amplicon data. Nat Methods. 2016, 13: 581–583. 10.1038/nmeth.3869 27214047 10.1038/nmeth.3869 PMC4927377

[35] Mcmurdie PJ., Holmes S. Phyloseq: A bioconductor package for handling and analysis of high-throughput phylogenetic sequence data. Pacific Symposium on Biocomputing. Pacific Symposium on Biocomputing, 2012, 235–246. 22174279 PMC3357092

[36] Pedersen, TL. ggforce: Accelerating 'ggplot2'.2018, Rpackage version 0.1.3. https://CRAN.R-project.org/package=ggforce

[37] Bandi C., Damiani G., Magrassi L., Grigolo A., Fani R., Sacchi L. Flavobacteria as intracellular symbionts in cockroaches. Proc Biol Sci. 1994, 257: 43–48. 10.1098/rspb.1994.0092 8090791 10.1098/rspb.1994.0092

[38] Bandi C., Sironi M., Damiani G., Magrassi L., Nalepa CA., Laudani U., Sacchi L.The establishment of intracellular symbiosis in an ancestor of cockroaches and termites. Proc Biol Sci. 1955, 259: 293–299. 10.1098/rspb.1995.0043 7740047

[39] O’Neill SL., Gooding RH., Aksoy R. Phylogenetically distant symbiotic microorganisms reside in Glossina midgut and ovary tissues. Med Vet Entomol. 1993, 7:377–383. 8268495 10.1111/j.1365-2915.1993.tb00709.x

[40] Kakumanu M., Maritz JM., Carlton JM., Schal C.Overlapping community composition of gut and fecal microbiomes in lab-reared and field-collected German cockroaches. Appl Environ Microbiol. 2018, 10.1128/AEM.01037-18 29959246 10.1128/AEM.01037-18 PMC6102980

[41] Tegtmeier D., Thompson CL., Schauer C., Brune A. Oxygen affects gut bacterial colonization and metabolic activities in a gnotobiotic cockroach mode. Appl Environ Microbiol. 2016, 82: 1080–1089. 10.1128/AEM.03130-15 26637604 10.1128/AEM.03130-15 PMC4751835

[42] Maurice CF., Haiser HJ., Turnbaugh PJ. Xenobiotics shape the physiology and gene expression of the active human gut microbiome. Cell. 2013, 152: 39–50. 10.1016/j.cell.2012.10.052 23332745 10.1016/j.cell.2012.10.052 PMC3552296

[43] Kane MD., Breznak JA. Effect of host diet on production of organic acids and methane by cockroach gut bacteria. Appl Environ Micrbiol. 1991, 57: 2628–2634. 10.1128/aem.57.9.2628-2634.1991 PMC183631 1662936

[44] Zhang J., Pan Y., Zheng C., Gao X., Wie X., Xi J., et al Rapid evolution of symbiotic bacteria populations in spirotetramat-resistant *Aphis gossypii* glover revealed by pyrosequencing. Comp Biochem Physiol. 2016, 20: 151–158. 10.1016/j.cbd.2016.10.001 27788413

[45] Ramya SL., Venkatesan KT, Srinivasa Murthy K., Jalali SK., Verghese A. Detection of carboxylesterase and esterase activity in culturable gut bacterial flora isolated from the diamondback moth *Plutella xylostella* (Linnaeus), from India and its possible role in indoxacarb degradation. Brazil J Microbiol. 2016, 47: 327–336. 10.1016/j.bjm.2016.01.012 PMC4874610 26991291

[46] Gondhalekar AD., Nakayasu ES., Silva I., Cooper B., Scharf ME. Indoxacarb biotransformation in the German cockroach. Pestic Biochem Physiol. 2016, 134: 14–23. 10.1016/j.pestbp.2016.05.003 27914535 10.1016/j.pestbp.2016.05.003

[47] Zhang S., Zhang X., Shen J., Li D., Wan H., You H., Li J. Cross-resistance and biochemical mechanisms of resistance to indoxacarb in the diamondback moth, *Plutella xylostella*. Pestic Biochem Physiol. 2017, 140:85–89. 10.1016/j.pestbp.2017.06.011 28755699 10.1016/j.pestbp.2017.06.011

[48] Soltani A., Vatandoost H., Oshaghi MA., Enayati AA., Chavshin AR. The role of midgut symbiotic bacteria in resistance of *Anopheles stephensi* (Diptera: Culucidae) to organophosphate insecticides. Pathog Glob Health. 2017, 111: 289–296. 10.1080/20477724.2017.1356052 28745553 10.1080/20477724.2017.1356052 PMC5694856

[49] Ayayee PA., Larsen T., Sabree Z. Symbiotic essential amino acids provisioning in the American cockroach, *Periplaneta Americana* (Linnaeus) under various dietary conditions. PeerJ. 2016, 4:e2046 10.7717/peerj.2046 27231663 10.7717/peerj.2046 PMC4878363

[50] Ayayee PA., Ondrejech A., Keeney G., Munoz-Garcia A. The role of the gut microbiota in the regulation of standard metabolic rate in female *Periplaneta Americana*. PeerJ. 2018, 6: e4717 10.7717/peerj.4717 29844953 10.7717/peerj.4717 PMC5971104

[51] Vaishampayan PA., Dhotre DP., Gupta RP., Lalwani P., Ghate H., Patole MS., Shouche YS. Molecular evidence and phylogenetic affiliations of Wolbachia in cockroaches. Mol Phylogenet Evol. 2007, 44: 1346–1351. 10.1016/j.ympev.2007.01.003 17350292 10.1016/j.ympev.2007.01.003

[52] Jensen K., Ko AE., Schal C., Silverman J. Insecticide resistance and nutrition interactively shape life-history parameters in in German cockroaches. Sci Rep. 2016, 6: 28731 10.1038/srep28731 27345220 10.1038/srep28731 PMC4922014

