# Supplementary figures and images for: Disruption of the microbiota affects physiological and evolutionary aspects of insecticide resistance in the German cockroach, an important urban pest
Source: PLoS One. 2018 Dec 12;13(12):e0207985. doi: 10.1371/journal.pone.0207985 (PMC6291076; doi:10.1371/journal.pone.0207985)

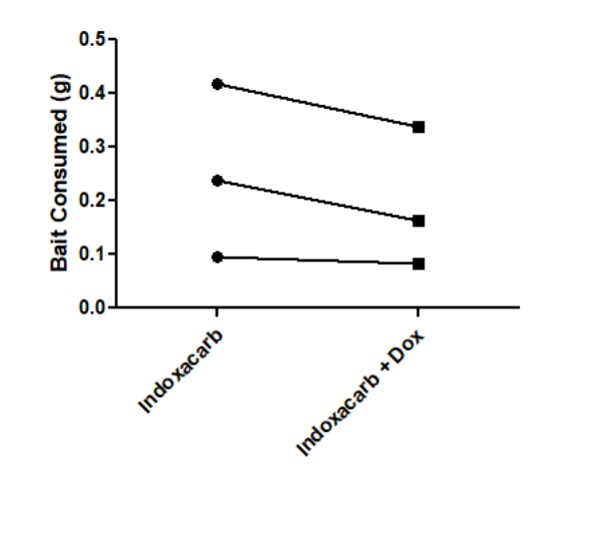

Supplement: S1 Fig — Customized gel baits containing indoxacarb alone (0.05%) or in combination with doxycycline (0.5%) were placed side-by-side in experimental arenas and consumption over a 4-hour period was determined by weight. The experiment was independently replicated 3 times using arenas with varied cockroach densities. (TIF) [file pone.0207985.s001.tif]

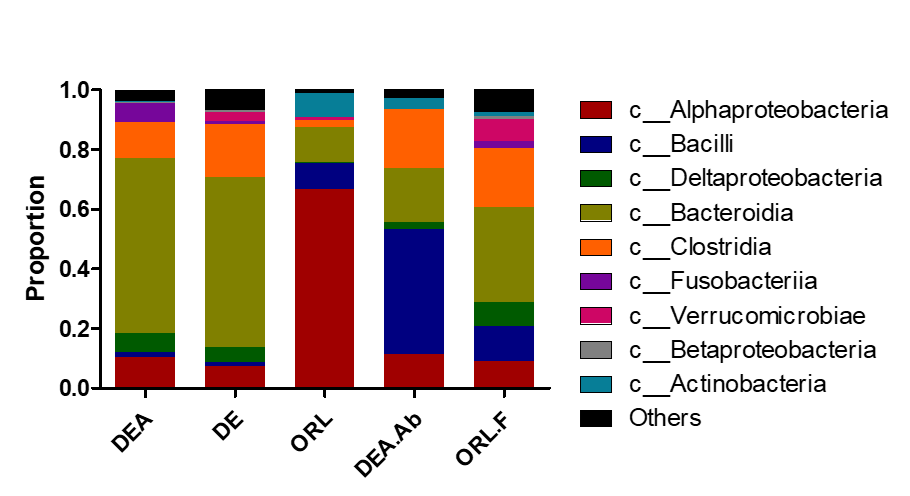

Supplement: S2 Fig — Reads assigned to the Class Gammaproteobacteria were excluded from analysis and the relative abundance of the remaining taxa was determined. (TIF) [file pone.0207985.s002.tif]

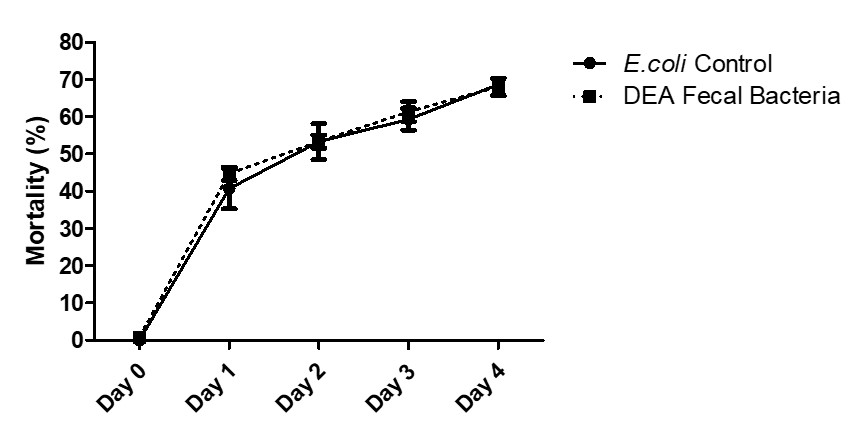

Supplement: S3 Fig — Cultures of E. coli (control) or fecal bacteria from the bait-selected, resistant strain (DEA) were grown overnight in liquid LB containing 1 mg/ml indoxacarb and used in place of water to formulate customized gel baits with a final indoxacarb concentration of 0.05%. Susceptible cockroaches (ORL) were exposed to baits and mortality was assessed over a period of 4 days. N = 3 replicates of 50 insects. (TIF) [file pone.0207985.s003.tif]

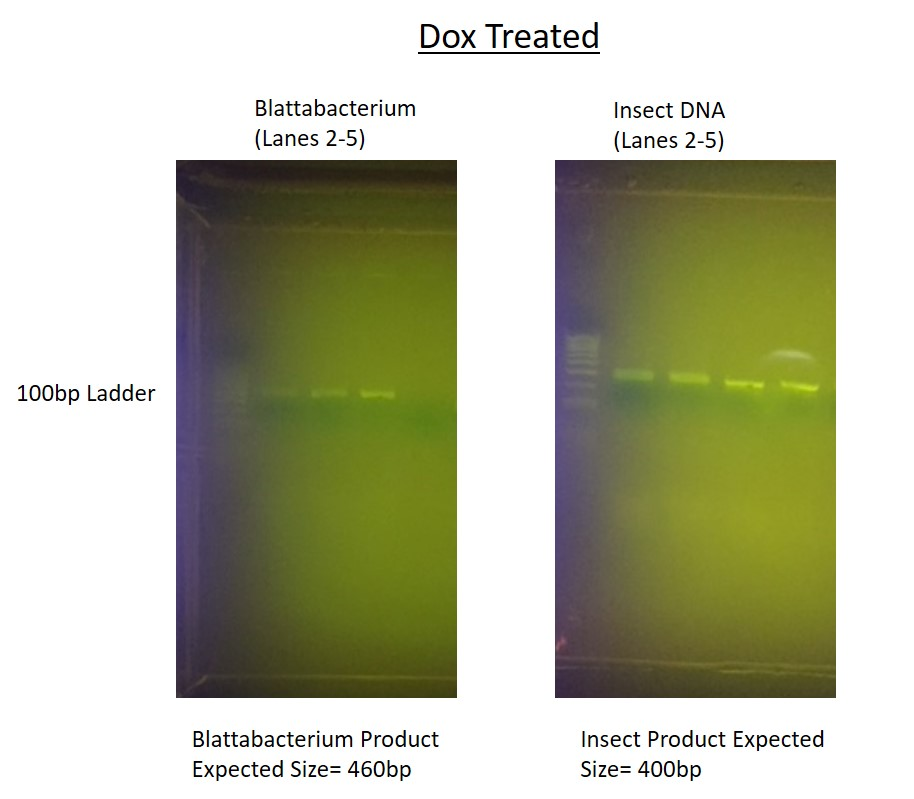

Supplement: S4 Fig — (TIF) [file pone.0207985.s004.tif]
