# Supplementary material for: Disruption of the microbiota affects physiological and evolutionary aspects of insecticide resistance in the German cockroach, an important urban pest
Source: PLoS One. 2018 Dec 12;13(12):e0207985. doi: 10.1371/journal.pone.0207985 (PMC6291076; doi:10.1371/journal.pone.0207985)
Supplement: S1 Table — (DOCX) [file pone.0207985.s005.docx]

| **Sample** | **Raw** | **Filtered** | **Denoised F** | **Denoised R** | **Merged** | **Non-Chimeric** |
| --- | --- | --- | --- | --- | --- | --- |
| DE | 96167 | 89969 | 87835 | 88531 | 78266 | 50211 |
| DEA | 100931 | 95335 | 93368 | 94144 | 88702 | 56387 |
| DEA.Ab | 109551 | 103527 | 101993 | 102699 | 98550 | 66971 |
| ORL | 91904 | 86492 | 85490 | 85925 | 83177 | 63551 |
| ORL.F | 113864 | 106402 | 104446 | 105283 | 88034 | 67916 |
