# Supplementary material for: Disruption of the microbiota affects physiological and evolutionary aspects of insecticide resistance in the German cockroach, an important urban pest
Source: PLoS One. 2018 Dec 12;13(12):e0207985. doi: 10.1371/journal.pone.0207985 (PMC6291076; doi:10.1371/journal.pone.0207985)
Supplement: S1 File — (DOCX) [file pone.0207985.s006.docx]

**Supplementary Materials & Methods**

**Bait consumption assays**

Consumption tests to determine bait palatability **(S1 Fig)** were performed using identical baits to those used for toxicity assays (see antibiotic and insecticide treatments). 1000-2000 susceptible (ORL) cockroaches of mixed stage and sex were placed into experimental areas under the previously described standard experimental conditions. Freshly made gel baits containing either indoxacarb alone (0.05%) or indoxacarb with doxycycline (0.5%) were weighed on small petri dished and placed side-by-side into the experimental areas. Cockroaches were allowed to feed on baits for 4 hours. After this time, baits were re-weighed. To determine total consumption, final weight measurements were standardized for water loss and subtracted from initial weight measurements.

**Culture of gut microbes**

Gut microbes were cultured by collecting 10-15 fecal pellets from a bait-selected, resistant cockroach colony (DEA), vortexing, and incubating them in liquid LB media on a shaker at 30°C overnight. To determine if gut bacteria could reduce the toxicity of indoxacarb, fecal cultures were also grown overnight in liquid LB containing 1 mg/ml indoxacarb **(S2 Fig)**. The overnight cultures were subsequently used in the place of water to formulate customized gel baits with a final indoxacarb concentration of 0.05% for determination of toxicity (see antibiotic and insecticide treatments). Susceptible cockroaches (ORL) were treated with these baits and mortality over a period of 4 days was compared to insects treated with a control bait made from an overnight LB culture of *E. coli* containing 1 mg/ml indoxacarb. Survival curves were analyzed by two-way ANOVA using GraphPad Prism 5.
